# Effectiveness of steroid-free immunosuppressive regimens after kidney transplantation: the pioneering experience of a brazilian center

**DOI:** 10.1590/2175-8239-JBN-2025-0062en

**Published:** 2025-09-22

**Authors:** Tainá Veras de Sandes-Freitas, Flávio Bezerra de Araújo, Raoni de Oliveira Domingues-da-Silva, Maria Luíza de Mattos Brito Oliveira Sales, Ronaldo de Matos Esmeraldo

**Affiliations:** 1 Universidade Federal do Ceará, Faculdade de Medicina, Fortaleza, Ceará, Brazil.; 2 Universidade Estadual do Ceará, Mestrado Profissional em Transplantes, Fortaleza, Ceará, Brazil.; 3 Hospital Geral de Fortaleza, Fortaleza, Ceará, Brazil.

**Keywords:** Kidney Transplantation, Immunosuppression Therapy, Steroids, Adrenal Cortex Hormones

## Abstract

**Introduction:**

Chronic corticosteroid use, even at low doses, is associated with well-known adverse effects. However, steroid-free regimens after kidney transplantation (KT) have been linked to a higher incidence of acute rejection (AR), limiting their implementation to a few centers worldwide. In this study, we describe the pioneering experience of a Brazilian center that adopted a steroid-free immunosuppressive regimen in 2005 for patients at low to moderate immunological risk.

**Methods:**

This single-center retrospective cohort study includes KT recipients who were submitted to steroid-free regimens between 2012 and 2019. The cohort was followed for three years in a real-world setting.

**Results:**

A total of 562 patients were included, 71.4% male, with a median age of 48.1 years (IQR 35.5–58.6). Most (95.2%) received deceased donor allografts. All patients underwent induction therapy with antithymocyte globulin, and 82.2% received tacrolimus in combination with sirolimus or everolimus as maintenance therapy. After three years, 10.2% experienced treated AR episodes, with biopsy confirmation in 3.2%. Age (HR 0.946, 95% CI 0.923–0.969, p < 0.001) and HLA mismatches (HR 1.312, 95% CI 1.021–1.687, p = 0.034) were risk factors for rejection. Twenty-eight patients (5%) lost their grafts, and 5.7% died. Seventy-one patients (12.6%) required corticosteroid introduction over the years, with a median of 125.5 days (IQR 31.7–409) post-KT. The main reasons were perceived immunosuppressive regimen inefficacy (56.3%) and composition in a low-efficacy immunosuppressive regimen (18.6%).

**Conclusion:**

Steroid-free immunosuppression was effective in low to moderate immunological risk KT recipients over a three-year follow-up period.

## Introduction

The standard maintenance immunosuppressive regimen for patients at low to moderate immunological risk consists of a combination of a calcineurin inhibitor (tacrolimus or cyclosporine), an antiproliferative agent (azathioprine, mycophenolate, sirolimus, or everolimus), and a corticosteroid^1^.

Prolonged steroid use is associated with a higher incidence of hypertension, dyslipidemia, diabetes, osteoporosis, fractures, excessive weight gain, and an increased risk of infections and cardiovascular events^2,3^. A large international cohort from the “Collaborative Transplant Study” (CTS) registry showed that patients receiving corticosteroids at doses as low as 0.01–0.04 mg/kg one year after KT had a higher risk of death from cardiovascular events and infections compared to individuals who were not receiving these drugs^4^.

To mitigate these risks, some centers worldwide have tested regimens that eliminate or avoid these drugs^5,6,7^. Clinical trials and observational studies analyzing these experiences have shown heterogeneous results, some of them reporting higher rates of acute rejection and poorer survival^8,9,10^. However, studies involving patients undergoing highly effective maintenance therapies, i.e., induction with rabbit antithymocyte globulin (rATG) and maintenance with the combination of tacrolimus and mycophenolate or tacrolimus and a mammalian target of rapamycin inhibitor (mTORi) have demonstrated efficacy in preventing acute rejection comparable to that of steroid-containing regimens, as well as similar survival^11,12^.

Despite these results, steroid-free regimens are not yet a reality worldwide. In the USA, for example, only about 25% of KT patients follow this strategy^13^. Since 2001, the *Hospital Geral de Fortaleza*, in Ceará, has implemented a steroid-free immunosuppression protocol for patients at low to moderate immunological risk, pioneering this approach in Latin America. The present study aimed to describe the outcomes of this experience over a 3-year follow-up period.

## Methods

### Study Design, Research Setting, and Study Population

Retrospective, single-center cohort study including adult patients who underwent isolated KT at the *Hospital Geral de Fortaleza*, Fortaleza, Ceará, from January 2012 to December 2019. All patients received a steroid-free maintenance immunosuppressive regimen. The following were excluded: HLA-identical living donor KT; patients who received exceptional maintenance regimens, i.e., those not based on the combination of tacrolimus with either an mTOR inhibitor or mycophenolate; death or graft loss within the first week after KT, without sufficient time to establish maintenance immunosuppression; and patients from other services who underwent transplantation surgery at our center and returned to their original center for follow-up within 90 days after KT.

### Evaluated Outcomes

The 3-year effectiveness outcomes were evaluated, including the incidence and severity of acute rejection episodes, graft loss and mortality rates, kidney function, as well as the occurrence and reasons for introducing corticosteroids into the immunosuppressive regimen during follow-up. Some adverse events commonly associated with corticosteroid use, such as diabetes, dyslipidemia, and weight gain, were also evaluated.

### Center Immunosuppression Protocol

According to the center’s protocol, patients considered to be at low to moderate immunological risk are candidates for steroid-free regimens: first transplant, negative complement-dependent cytotoxicity (CDC) crossmatch against T and B lymphocytes, panel reactive antibody (PRA) below 50% and absence of donor-specific anti-HLA antibodies (DSA) in either historical or current serum, as determined by the Luminex® method, One Lambda Single Antigen platform, defined by the presence of anti-HLA A, B, DR, or DQ DSA with a mean fluorescence intensity (MFI) greater than 1500. The thresholds for risk stratification, based on these tests, were applied consistently throughout the study period. The center did not perform desensitization treatments or ABO-incompatible KT during the study period.

All patients received induction therapy with rabbit antithymocyte globulin (rATG). From January 2012 to December 2018, the total dose used was 6 mg/kg (divided into four doses of 1.5 mg/kg on alternate days, with the first dose administered intraoperatively before declamping the anastomoses). Since January 2019, the recommended standard dose has been 4.5 mg/kg (three doses of 1.5 mg/kg, the first administered intraoperatively, the second immediately afterward, and the third on postoperative day two). Lymphocyte counts are routinely performed; however, they are not used as a parameter for rATG dose adjustment. Methylprednisolone 250 mg is administered before the first rATG dose and, therefore, prior to graft revascularization. Subsequent doses of rATG are preceded by 125 mg of methylprednisolone, and when a fourth dose of rATG is necessary, a 20 mg prednisone dose is administered as premedication.

Standard maintenance immunosuppression consisted of the combination of tacrolimus (target trough concentration of 4–6 ng/mL) and an mTOR inhibitor - sirolimus or everolimus (both with a target trough concentration of 4–6 ng/mL). Patients with contraindications to the use of mTOR inhibitors or, in special cases, at the discretion of the attending team - such as in glomerulopathies – received mycophenolate sodium at an initial dose of 1440 mg/day, with subsequent adjustments depending on the occurrence of adverse events. Azathioprine is not used as part of the center’s initial immunosuppressive regimen.

Intravenous corticosteroids (methylprednisolone) or oral corticosteroids (prednisone or prednisolone) were administered exclusively as premedication prior to rATG infusions, as previously outlined. Subsequent to this period, no additional doses were administered with the objective of composing the maintenance immunosuppressive regimen.

There was no predetermined protocol for monitoring patients, including protocol biopsies or routine requests for DSA investigation. Similarly, no predefined protocol was implemented for post-KT corticosteroid administration, with this decision being delegated to the attending physician based on their perception and weighing of risks and benefits. There were also no preemptive conversions of the immunosuppressive regimen. All conversions were driven by concerns regarding the regimen’s efficacy or safety in a real-world setting, based on the perception of the attending clinical team.

### Definitions

Delayed graft function (DGF) was defined as the need for dialysis in the first week post-transplantation, considering and disregarding isolated sessions performed within the first 24 hours after the procedure, due to hyperkalemia and/or hypervolemia^14^. The duration of DGF was assessed as the interval, in days, between the KT and the last dialysis session.

Expanded criteria donor (ECD) was defined as individuals aged over 60 years; or aged between 50 and 59 years and meeting at least two of the following three criteria: history of hypertension, stroke as cause of death, or final creatinine greater than 1.5 mg/dL^15^.

Post-transplant diabetes mellitus (PTDM) was defined as the need for treatment with oral antidiabetic agents or insulin after KT in patients without a previous diagnosis of diabetes^16^.

Statin therapy was initiated at the discretion of the physician, based on lipid levels and cardiovascular risk, without a pre-established protocol for its prescription.

Treated acute rejection (AR) was defined as an episode of graft dysfunction that resulted in treatment with methylprednisolone pulse therapy or rATG, plasmapheresis sessions, and/or immunoglobulin administration, regardless of whether a biopsy was performed or findings were present. Biopsy-proven acute rejection (BPAR) was defined as an AR episode in which biopsy showed antibody-mediated rejection (AMR) or acute cellular rejection grade IA or higher, according to the Banff classification in effect at the time of biopsy^17^.

Renal function was assessed using serum creatinine and estimated glomerular filtration rate (eGFR), calculated according to the CKD-EPI 2021 formula^18^.

We grouped under the term “perceived therapeutic ineffectiveness” – as a reason for corticosteroid introduction – episodes of treated AR, the emergence of *de novo* DSA, or increased PRA during follow-up.

### Statistical Analysis

Categorical variables were described using absolute frequencies and percentages and compared using the Chi-square test or Fisher’s exact test, when applicable. The Shapiro-Wilk test was used to verify the normality of distribution of numerical variables. Numerical variables with a normal distribution were expressed as mean and standard deviation; those with a nonparametric distribution were expressed as median and interquartile range (IQR). Survival curves were constructed using the Kaplan Meier method. In the graft survival analysis, death was censored.

In the estimated glomerular filtration rate (eGFR) analysis, missing data were imputed using the Last Observation Carried Forward (LOCF) method, assigning a value of zero to patients who lost the graft and the last available value to those who died or were lost to follow-up^19^. For the other numerical and categorical variables, no imputation of missing values was performed.

Multivariate analysis to identify independent risk factors for rejection was performed using Cox regression. Variables with a p-value <0.1 in the univariate analysis were included in the multivariate model. In both the multivariate analysis and other analyses, results with a p-value <0.05 were considered significant.

### Ethical Aspects

The study followed Resolution 466/12 of the Brazilian National Health Council for research involving human subjects, and was submitted to the Research Ethics Committee of the *Hospital Geral de Fortaleza*. It was approved in accordance with opinion no. 5.486.193 and CAAE no. 59301622.0000.5040.

## RESULTS

### Sample and Clinical-Demographic Characteristics

**Figure 1:**
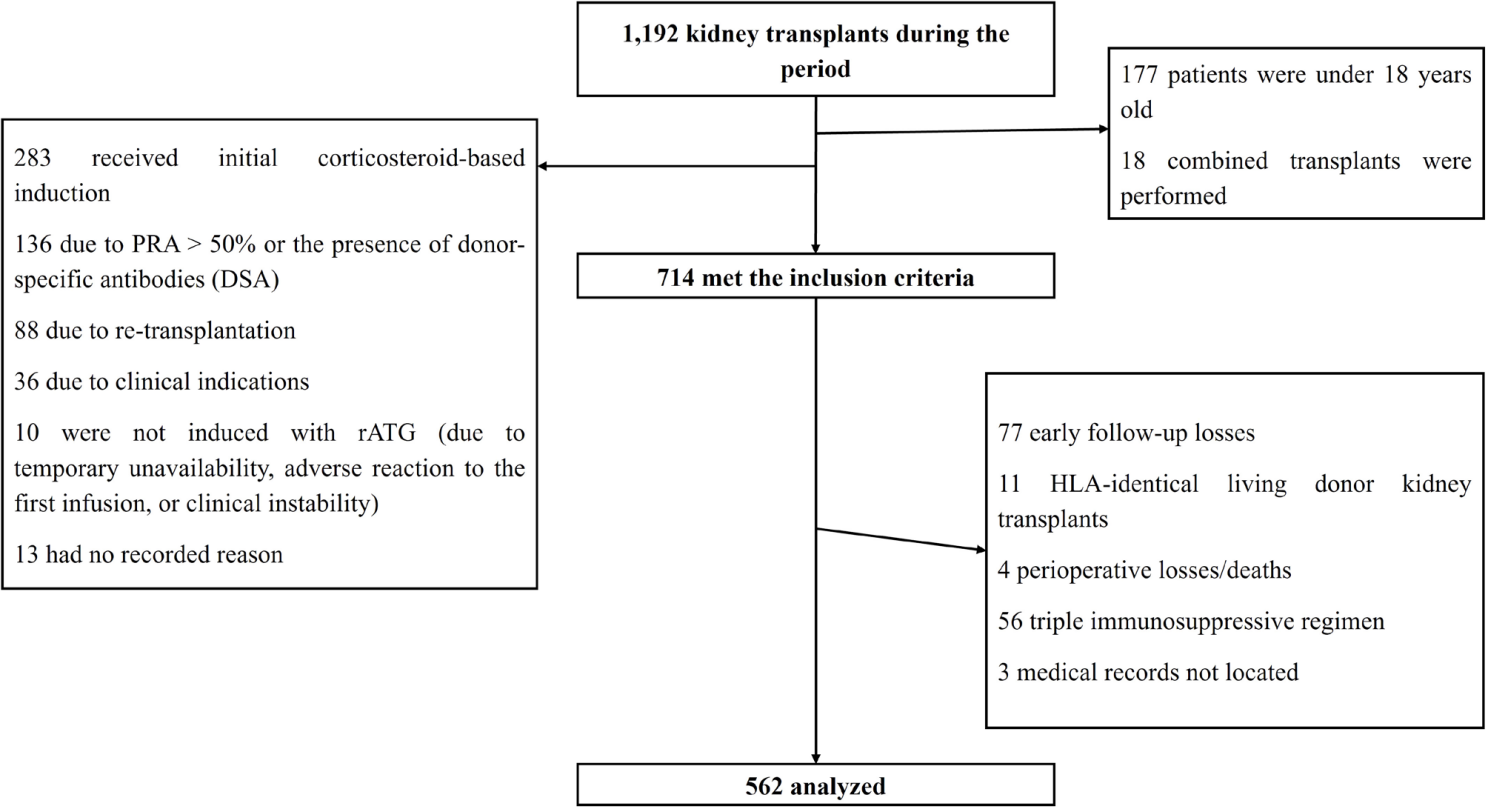
Sample definition.

Of the 1,192 kidney transplants performed during the study period, 714 initially met the inclusion criteria. After applying the exclusion criteria - including 4 deaths or graft losses due to surgical complications in the first week and 77 early losses to follow-up - 562 patients were included in the final analysis (Figure 1).

Abbreviations – DSA: donor-specific anti-HLA antibodies; HLA: human leukocyte antigen; IS: immunosuppressant; PRA: panel reactive antibody; rATG: rabbit antithymocyte globulin; KT: kidney transplant.

Patients were predominantly male (71.4%) and of mixed race (74.6%), with a median age of 48.1 years (IQR 35.5–58.6). The main etiology of kidney disease was undetermined (38.1%), followed by diabetes (19.6%), and glomerulopathies (14.1%). Nearly all KT were performed with deceased donors (95.2%), with only 6.6% coming from ECD. Table 1 details the demographic and clinical characteristics of patients at the time of KT.

**Table 1:** Demographic and clinical characteristics of the sample

Variable	N	Result
Recipient age (years), median (IQR)	562	48.1 (35.5-58.6)
Male, n(%)	562	401 (71.4)
Skin color, n(%) *Brown* *White* *Black* *Red* *Yellow*	562	419 (74.6) 84 (14.9) 57 (10.1) 1 (0.2) 1 (0.2)
BMI (Kg/m^2^), median (IQR)	559	24.5 (22.0–27.7)
Etiology of CKD, n(%) *Undetermined* *DM* *CGN* *SAH* *ADPKD* *Urological* *Others*	562	214 (38.1) 110 (19.6) 79 (14.1) 62 (11.0) 48 (8.5) 26 (4.6) 23 (4.1)
History of DM, n(%)	562	118 (21.0)
Dialysis vintage (months), median (IQR)	561	32 (18–59)
Positive serology, n(%) *Hepatitis B* *Hepatitis C* *HIV*	562	5 (0.9) 6 (1.1) 4 (0.7)
PRA I (%), median (IQR) PRA I 1 - 50%, n(%)	562	0 (0–0) 89 (15.8)
PRA II (%), median (IQR) PRA II 1 - 50%, n(%)	562	0 (0–0) 29 (5.2)
Donor type, n(%) *Deceased* *Living HLA haploidentical* *Living HLA mismatched*	562	535 (95.2) 14 (2.5) 13 (2.3)
Donor age, median (IQR) ECD, n(%)*	535 535	32 (23–42) 37 (6.6)
Machine perfusion, n(%)*	535	271 (48.2)
HLA MM, median (IQR)*	535	3 (2–4)
MMDR, n(%)* *0* *1* *2*	535	197 (35.1) 261 (46.4) 77 (13.7)
CIT (h), median (IQR)*	530	22.0 (18.7–26.0)

Abbreviations – IQR: Interquartile range; BMI: Body mass index; CKD: Chronic kidney disease; DM: Diabetes mellitus; CGN: Chronic glomerulonephritis; SAH: Systemic arterial hypertension; ADPKD: Autosomal dominant polycystic kidney disease; ECD: Expanded criteria donor; MM: Mismatch; CIT: Cold ischemia time.

### Immunosuppression

The median thymoglobulin dose administered was 5.5 mg/kg (IQR 4.3–6.0). Throughout the study period, the total rATG dose ranged from 3 mg/kg to 6 mg/kg, according to protocol changes and participation in clinical trials, with 4.5 mg/kg being the most recently standardized dose for this risk group.

**Figure 2:**
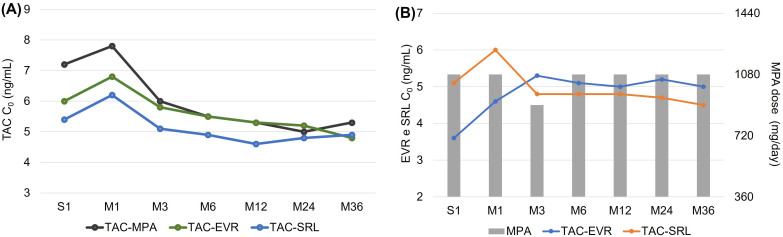
Exposure to immunosuppressants over a 3-year period.

Regarding maintenance regimens, 44.5% of patients received a combination of tacrolimus and everolimus, 37.7% tacrolimus and sirolimus, and 17.8% tacrolimus and mycophenolate. Tacrolimus and mTOR inhibitor concentrations, as well as the median mycophenolate dose throughout follow-up, are depicted in Figure 2.

Abbreviations – C_0_: trough blood concentration; EVR: everolimus; MPA: mycophenolate; SRL: sirolimus; TAC: tacrolimus. Notes - (A) Trough blood concentration of tacrolimus (ng/mL); trough blood concentration of sirolimus and everolimus (ng/mL); and mean dose of mycophenolate (mg/day).

### Safety, Effectiveness, Renal Function, and Survival Outcomes

Among the safety outcomes investigated, the incidence of PTDM was observed in 21.3% of patients, and 4.7% required insulin treatment. More than half of the patients (54.9%) were using statins at the end of 3 years, and the median weight gain was 3.5 kg (IQR –0.5 to +7.6).

Among the deceased-donor transplants, 42.3% presented DGF, with a median duration of 8 days (IQR 3–17). When isolated dialysis sessions performed in the perioperative period were excluded, the incidence was 35.5%.

The incidence of treated acute rejection was 10.2%, with most treated episodes not undergoing biopsy or, when performed, presenting borderline or marginal infiltrates. The median time to the first episode of treated AR was 26 days (IQR 12–156.5 days). Forty-three of the 57 patients (75.4%) received methylprednisolone for the treatment of AR episodes, 9 (15.8%) were treated with rATG, and 5 (8.8%) with plasmapheresis and/or immunoglobulin.

Considering only biopsy-proven rejections, the incidence was 3.2%, with 8 of the 18 episodes characterized as mild cellular rejections, with a median time to occurrence of 39 days (IQR 13.75–170.5 days).

Patient and graft survival rates at the end of the 3-year follow-up period were 94.1% and 94.9%, respectively. Table 2 details the 3-year outcomes.

**Table 2:** Safety and effectiveness outcomes at 3 years

Variable	N	Result
PTDM, n(%)	423	90 (21.3)
PTDM requiring insulin, n(%)	423	20 (4.7)
Need for statins, n(%)	532	292 (54.9)
Weight gain (kg), median (IQR)	478	+3.5 (–0.5 – +7.6)
DGF, n(%)*	532	225 (42.3)
DGF excluding isolated dialysis in IPO/PO day 1, n(%)*	532	189 (35.5)
Time in DGF (days), median (IQR)*	224	8 (3–17)
Treated AR, n(%)	559	57 (10.2)
*RB not performed* *No AR* *Borderline infiltrate* *Banff IA* *Banff IB* *Banff IIA* *Banff AMR*		18 1 20 8 2 5 3
BPAR, n(%) BPAR + borderline infiltrates, n(%)	559 559	18 (3.2) 38 (6.8)
Treated AR-free survival at 3 years, n(%)	560	454 (89.6)
BPAR-free survival at 3 years, n(%)	560	488 (96.7)
Graft loss, n(%) *Non-immune IFTA* *Acute rejection* *Primary graft failure* *Vascular thrombosis* *Post-biopsy complication* *Urinary fistula* *Recurrent or de novo GN* *Renal artery pseudoaneurysm* *Cortical necrosis* *BKV nephropathy* *Acute pyelonephritis* *Kidney infection*	562	28 (5.0) 5 3 3 3 3 3 2 2 1 1 1 1
Graft survival at 3 years, n(%)	562	502 (94.9)
Death, n(%) *Infection* *Neoplasm* *Cardiovascular event* *Pancreatitis* *Hemorrhagic shock* *Unknown cause*	562	32 (5.7) 20 4 4 2 1 1
Patient survival at 3 years, n(%)	562	501 (94.1)
eGFR at 3 years (mL/min/1.73m^2^), median (IQR)	500	65.0 (49.5–82.7)
eGFR at 3 years with LOCF adjustment (mL/min/1.73m^2^), median (IQR)	561	62.0 (46.4–81.1)

Abbreviations – PTDM: Post-transplant diabetes mellitus; IQR: Interquartile range; DGF: Delayed graft function; AR: Acute rejection; RB: Renal biopsy; BPAR: Biopsy-proven acute rejection; IFTA: Interstitial fibrosis and tubular atrophy; GN: Glomerulonephritis; GFR: Glomerular filtration rate; LOCF: Last observation carried forward.

In the multivariate analysis, recipient age (HR 0.946; 95%CI 0.923–0.969; p < 0.001) and HLA mismatches (HR 1.312; 95%CI 1.021-1.687; p = 0.034) were the variables independently associated with the occurrence of BPAR (including borderline infiltrates) (Table 3).

**Table 3:** Risk factors for biopsy-proven acute rejection at 3 years

	Univariate			Multivariate N = 35*		
	HR	95%CI	P-value	HR	95%CI	P-value
Recipient age (years)	0.953	0.931–0.976	<0.001	**0.946**	**0.923–0.969**	**<0.001**
Male	0.771	0.394–1.507	0.447	NA		
Black	1.366	0.533–3.499	0.516	NA		
BMI (Kg/m2)	0.928	0.858–1.005	0.065	0.984	0.960–1.069	0.709
History of DM	0.205	0.049–0.853	0.029	0.351	0.081–1.522	0.162
Dialysis vintage (months)	1.003	0.996–1.010	0.388	NA		
Deceased donor	0.562	0.173–1.828	0.338	NA		
Donor age (years)	1.528	0.763–3.060	0.232	NA		
HLA MM	1.368	1.045–1.790	0.022	**1.312**	**1.021**–**1.687**	**0.034**
PRA I (%)	1.021	0.993–1.050	0.145	NA		
PRA II (%)	0.993	0.932–1.059	0.839	NA		
rATG dose (mg/Kg)	0.769	0.597–0.992	0.044	0.876	0.673–1.142	0.328
Initial maintenance IS with mTOR inhibitor (vs. MPA)	0.963	0.424–2.186	0.963	NA		
DGF disregarding isolated dialysis in IPO/PO day 1	1.458	0.746–2.848	0.270	NA		

Abbreviations – BMI: Body mass index; DM: Diabetes mellitus; MM: Mismatch; ATG: Antithymocyte globulin; IS: Immunosuppression; MPA: Mycophenolate; DGF: Delayed graft function; PO: Postoperative.

### Corticosteroid Introduction

**Figure 3:**
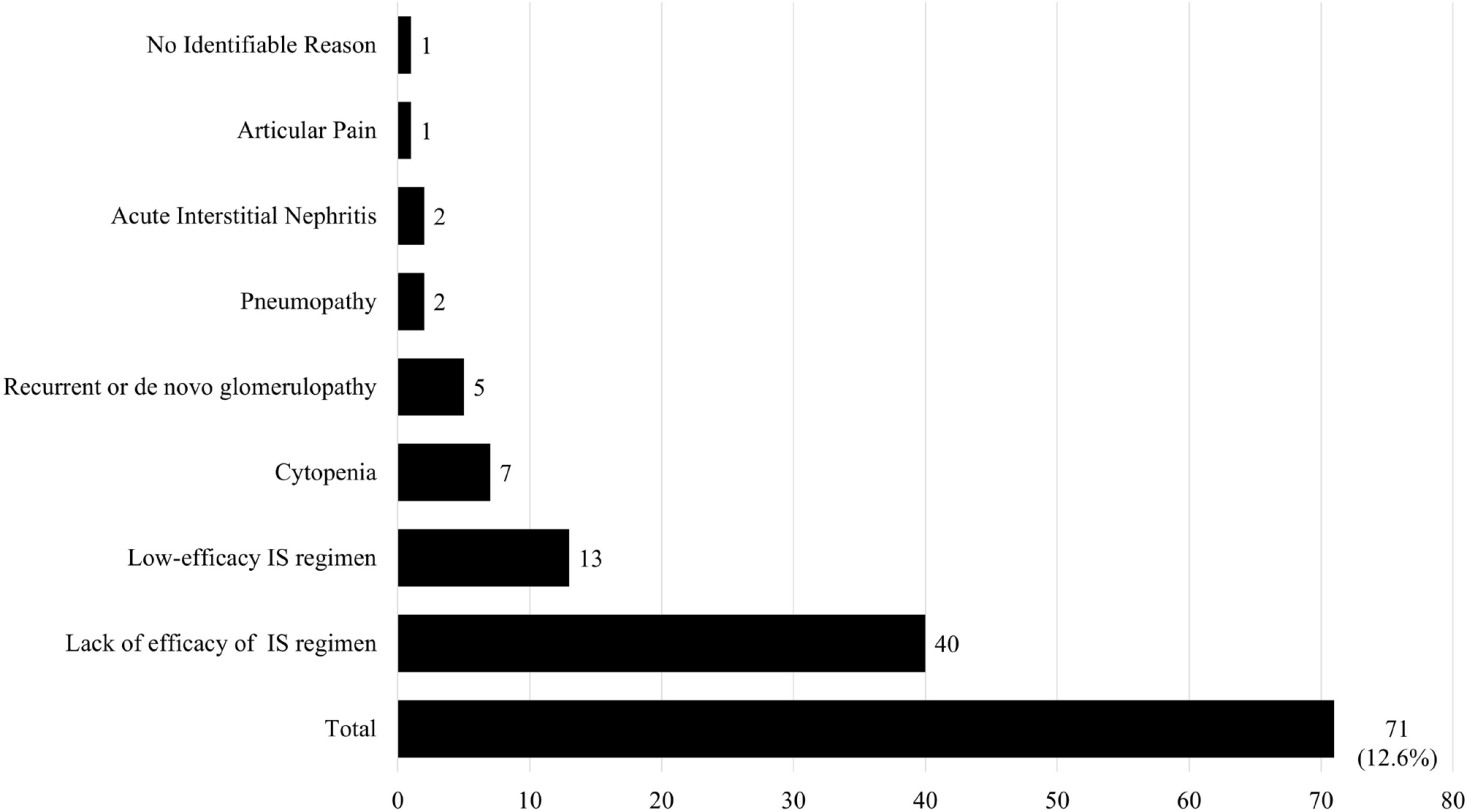
Need for corticosteroid introduction over the 3-year follow-up period.

During follow-up, 71 patients (12.6%) required the introduction of corticosteroids into the maintenance regimen, with most introductions occurring in the first months after KT, at a median of 125.5 days (IQR 31.7-409). The main reasons were the perceived therapeutic ineffectiveness of the regimen and the inclusion in an immunosuppressive regimen considered to be less effective (Figure 3). Among the 40 cases considered as perceived regimen failure, 34 corresponded to episodes of treated acute rejection, 5 to the emergence of DSA, and 1 case to increased PRA values without detectable DSA.

Of the 57 patients with treated AR, 22 did not initiate corticosteroids after the episode. In 18 of these cases, there was no evidence of rejection confirmed by biopsy (8 borderline infiltrates and 10 not biopsied). Among the 4 patients with BPAR, 1 lost the graft due to the rejection episode and 1 due to early surgical complications, preventing the subsequent introduction of corticosteroids. In the remaining 2 patients with BPAR, steroids were not initiated, and it was not possible to identify the reason based on records.

Of the 13 cases in which corticosteroids were introduced to compose a less effective regimen, 5 involved a combination of tacrolimus and azathioprine, 3 consisted of regimens with tacrolimus alone, 1 consisted of a regimen with mycophenolate alone, and in 4 cases, corticosteroids were maintained as the sole immunosuppressant.

## Discussion

This study demonstrated that the use of steroid-free immunosuppressive regimens based on rATG induction and initial maintenance therapy with tacrolimus and mycophenolate or mTOR inhibitor was effective in preventing episodes of acute rejection and had a good safety profile at a 3-year follow-up.

These findings are consistent with previously published single- and multicenter studies demonstrating that steroid-free regimens are effective in patients at low to moderate immunological risk when high-efficacy maintenance therapies are used – i.e., the combination of tacrolimus and mycophenolate or mTORi and rATG induction^20,21 22,23^. Our data add information on a subgroup that has been little explored in the literature, namely patients receiving tacrolimus in combination with an mTOR inhibitor.

In addition, although we did not perform a direct comparison of outcomes between the subgroups receiving mTORi versus mycophenolate, in the multivariate analyses, the type of initial antiproliferative drug did not emerge as an independent predictor for the occurrence of BPAR.

Notably, one of the challenges for the routine implementation of a steroid-free strategy is establishing the target doses and concentrations of the drugs that can ensure efficacy while minimizing adverse events, particularly nephrotoxicity. In this regard, the demographics of our sample likely constitute a crucial factor in ensuring the good results observed, since transplant recipients from young donors are less susceptible to adverse events related to over- or underexposure to immunosuppressive drugs^24^.

Another challenge is identifying which patients clearly benefit from this strategy and in which cases it should be avoided. The incidence of PTDM, statin use, and weight gain were similar to those observed in cohorts using steroids^25,26,27,28^. The lack of a control group subjected to steroid-based immunosuppression prevented us from identifying whether the strategy provided real benefits to the sample. Other adverse events associated with chronic steroid use, such as cataracts, osteoporosis, infections, and non-fatal cardiovascular events, were not evaluated.

The low rejection rate is noteworthy, even considering all treated episodes, with or without biopsy confirmation, as well as good renal function and low graft loss and mortality rates at 3 years. It should be noted that this is a cohort of young patients at low immunological risk who predominantly received kidneys from standard criteria donors, with almost half of them perfused using machine perfusion, a factor evidenced by the incidence of DGF significantly lower than the national average^29^. Within this low immunological risk subgroup, the multivariate analysis indicates that patients who were younger and had a higher number of HLA mismatches presented a higher risk of rejection. Similarly, the lack of a control group receiving corticosteroids prevents us from drawing more robust conclusions regarding the regimen’s efficacy. It is also unclear to what extent it is possible to advance within the group of patients at higher immunological risk.

It is also noteworthy that 71% of patients were male. National and local evidence points to unequal access to transplantation among women^30^. However, this percentage of men exceeds the figure reported in previous cohorts from our region (around 60%)^31,32^, which may be related to the higher proportion of women who are sensitized or have autoimmune diseases requiring baseline steroid use^33^.

A small proportion of patients (12.6%) had corticosteroids introduced into their regimen over the years, which occurred within the first few months, with most cases motivated by concerns over effectiveness - a factor that reflects the main reason for the hesitation of centers in implementing the strategy on a routine basis.

This study has some limitations that should be highlighted. Primarily, the lack of a control group prevents definitive conclusions regarding the effectiveness and safety of the strategy. Since the steroid-free regimen is the practice adopted at the center for patients at low to moderate immunological risk, we do not have a control group that is comparable from a demographic and clinical standpoint. The use of a control group from another center could introduce significant biases due to differences in demographics and clinical practices. Furthermore, this study was conducted at a single center located in a region with unique transplant demographics, with a low rate of expanded criteria donor utilization and an incidence of DGF below the national average. The exclusion of patients who lost the graft or died within the first week of transplantation may have potentially added some selection bias. We have no information on regular PRA monitoring, and no protocol biopsies were performed. We also did not explore whether this simplified regimen could increase adherence to immunosuppressants. Despite these limitations, this study presents real-world data, with a robust sample, and describes the results of a pioneering experience in Brazil and Latin America, in addition to adding information on the steroid-free strategy in patients receiving tacrolimus and mTOR inhibitors, for which evidence remains scarce.

In conclusion, in this cohort of patients at low to moderate immunological risk, the steroid-free maintenance regimen demonstrated a favorable effectiveness and safety profile at the end of 3 years after KT.

## Data Availability

The database supporting the results of this study is available upon reasonable request to the corresponding author, Taina Veras de Sandes-Freitas.
