# Supplementary material for: Effectiveness of steroid-free immunosuppressive regimens after kidney transplantation: the pioneering experience of a brazilian center
Source: J Bras Nefrol. 2025 Sep 22;47(4):e20250062. doi: 10.1590/2175-8239-JBN-2025-0062en (PMC12456451; doi:10.1590/2175-8239-JBN-2025-0062en)
Supplement: Translation

# Translation: Efetividade de regimes imunossupressores livres de corticoides após o transplante renal: a experiência pioneira de um centro brasileiro

**DOI:** 10.1590/2175-8239-JBN-2025-0062pt

**Authors:** Tainá Veras de Sandes-Freitas, Flávio Bezerra de Araújo, Raoni de Oliveira Domingues-da-Silva, Maria Luíza de Mattos Brito Oliveira Sales, Ronaldo de Matos Esmeraldo

**Affiliations:** 1 Universidade Federal do Ceará, Faculdade de Medicina, Fortaleza, Ceará, Brasil.; 2 Universidade Estadual do Ceará, Mestrado Profissional em Transplantes, Fortaleza, Ceará, Brasil.; 3 Hospital Geral de Fortaleza, Fortaleza, Ceará, Brasil.

**Keywords:** Transplante de Rim, Terapia de Imunossupressão, Esteroides, Corticosteroides

## Abstract

**Introdução:**

O uso crônico de corticoides, mesmo em baixas doses, está associado a efeitos indesejáveis bem conhecidos. Entretanto, regimes livres de corticoides após o transplante renal (TxR) foram associados a uma maior incidência de rejeição aguda (RA), limitando sua implementação a poucos centros no mundo. Neste estudo, descrevemos a experiência pioneira de um centro brasileiro que, desde 2005, adotou o regime imunossupressor livre de corticoides para pacientes de baixo a moderado risco imunológico.

**Métodos:**

Coorte retrospectiva, unicêntrica, incluindo receptores de TxR submetidos a regimes livres de corticoides entre 2012 e 2019. A coorte foi seguida por 3 anos em um cenário de vida real.

**Resultados:**

Foram incluídos 562 pacientes, 71,4% homens, 48,1 anos (IIQ 35,5–58,6), 95,2% submetidos a TxR com doador falecido. Todos induzidos com globulina antitimócito e 82,2% receberam tacrolimo associado a sirolimo ou everolimo como regime de manutenção. Após 3 anos, 10,2% apresentaram episódio de RA tratada e 3,2% tinham confirmação por biópsia. Idade (HR 0,946; IC95% 0,923–0,969; p < 0,001) e incompatibilidades HLA (HR 1,312; IC95% 1,021–1,687; p = 0,034) foram os fatores de risco para rejeição. Vinte e oito pacientes (5%) perderam o enxerto, 5,7% foram a óbito. Setenta e um (12,6%) necessitaram introduzir corticoide ao regime ao longo dos anos, com uma mediana de 125,5 dias (IIQ 31,7–409) após o TxR. Os principais motivos foram percepção de ineficácia terapêutica (56,3%) e composição em regime imunossupressor de menor eficiência (18,6%).

**Conclusão:**

A imunossupressão sem corticoides mostrou-se efetiva em receptores de TxR de baixo a moderado risco em 3 anos.

## Introdução

O regime imunossupressor de manutenção padrão para pacientes de baixo a moderado risco imunológico consiste na combinação de um inibidor de calcineurina (tacrolimo ou ciclosporina), um agente antiproliferativo (azatioprina, micofenolato, sirolimo ou everolimo) e um corticoide^1^.

O uso prolongado de esteroides está associado a maior ocorrência de hipertensão, dislipidemia, diabetes, osteoporose, fraturas, ganho excessivo de peso, além de maior risco de infecções e eventos cardiovasculares^2,3^. Uma grande coorte internacional, oriunda do registro *Collaborative Transplant Study* (CTS), demonstrou que pacientes em uso de doses tão baixas quanto 0,01–0,04 mg/kg de corticoide, 1 ano após o TxR, apresentaram maior risco de morte por eventos cardiovasculares e infecções, quando comparados a indivíduos que não faziam uso de tais drogas^4^.

Como forma de mitigar esses riscos, alguns centros ao redor do mundo testaram o uso de regimes que eliminam ou evitam esses fármacos^5-7^. Os ensaios clínicos e estudos observacionais que analisaram essas experiências demonstram resultados heterogêneos, alguns deles com maior ocorrência de rejeição aguda e pior sobrevida^8–10^. Entretanto, estudos envolvendo pacientes em uso de terapias de manutenção de alta eficácia, ou seja, indução com globulina antitimócito (rATG) e manutenção com a combinação de tacrolimo e micofenolato ou de tacrolimo e um inibidor *da mammalian target of rapamycin* (imTOR) evidenciaram eficácia na prevenção da rejeição aguda semelhante à dos regimes com corticoides, bem como sobrevida similar^11,12^.

Apesar desses resultados, o uso de regimes livres de corticoides ainda não é uma realidade ao redor do mundo. Nos Estados Unidos, por exemplo, somente cerca de 25% dos TxR adotam essa estratégia^13^. Desde 2001, o Hospital Geral de Fortaleza, no Ceará, implementou um protocolo de imunossupressão livre de corticoides para pacientes de baixo a moderado risco imunológico, configurando uma experiência pioneira na América Latina. O presente estudo teve como objetivo descrever os desfechos, ao longo de 3 anos, dessa experiência.

## Métodos

### Delineamento, Local da Pesquisa e População do Estudo

Estudo de coorte retrospectiva, unicêntrico, incluindo pacientes adultos submetidos a TxR isolado no Hospital Geral de Fortaleza, Fortaleza, Ceará, de janeiro de 2012 a dezembro de 2019, que receberam regime imunossupressor de manutenção livre de corticoides. Foram excluídos: TxR com doador vivo HLA idêntico; pacientes que receberam regimes de manutenção de exceção, ou seja, que não foram baseados na associação entre tacrolimo e um imTOR ou micofenolato; óbito ou perda de enxerto na primeira semana após o TxR, sem tempo hábil para definição da imunossupressão de manutenção; e pacientes provenientes de outros serviços, submetidos à cirurgia de transplante em nosso centro, e que retornaram para seguimento no centro de origem dentro de 90 dias após o TxR.

### Desfechos Avaliados

Foram avaliados os desfechos de efetividade em 3 anos, incluindo a incidência e a gravidade dos episódios de rejeição aguda, as taxas de perda do enxerto e óbito, a função renal, bem como a ocorrência e os motivos para a introdução do corticoide ao regime imunossupressor ao longo do acompanhamento. Também foram avaliados alguns eventos adversos comumente associados ao uso de corticoides, tais como diabetes, dislipidemia e ganho de peso.

### Rotina de Imunossupressão do Centro

De acordo com a rotina do centro, são candidatos a regimes livres de corticoides os pacientes considerados de baixo a moderado risco imunológico: primeiro transplante, prova cruzada por citotoxicidade dependente do complemento (CDC) contra linfócitos T e B negativa, painel de reatividade de anticorpos (PRA) inferior a 50% e ausência de anticorpos anti-HLA específicos contra o doador (DSA) no soro histórico ou atual, determinados pelo método de Luminex®, plataforma One Lambda *Single Antigen,* definidos pela presença de DSA anti-HLA A, B, DR ou DQ com intensidade média de fluorescência (MFI) superior a 1500. Os limiares para estratificação de risco, baseados nesses testes, foram aplicados de forma consistente durante todo o período do estudo. O centro não realizou tratamentos de dessensibilização nem TxR com incompatibilidade ABO no período avaliado.

Todos os pacientes foram induzidos com globulina antitimócito de coelho (rATG). De janeiro de 2012 a dezembro de 2018, utilizou-se a dose total de 6 mg/kg (dividida em quatro doses de 1,5 mg/kg, em dias alternados, sendo a primeira iniciada no intraoperatório, antes do desclampe das anastomoses). Desde janeiro de 2019, a dose padrão preconizada é de 4,5 mg/kg (três doses de 1,5 mg/kg, sendo a primeira administrada no intraoperatório, a segunda imediatamente após e a terceira no segundo dia de pós-operatório). A contagem de linfócitos é realizada rotineiramente, porém não é utilizada como parâmetro para ajuste da dose de rATG. Metilprednisolona 250mg é administrada antes da primeira dose de rATG e, portanto, antes da revascularização do enxerto. As doses subsequentes de rATG são precedidas por 125 mg de metilprednisolona e, quando a quarta dose de rATG se faz necessária, uma dose de prednisona de 20mg é administrada como pré-medicação.

A imunossupressão de manutenção padrão consistiu na combinação de tacrolimo (concentração residual alvo de 4-6 ng/mL) e um imTOR - sirolimo ou everolimo (ambos com concentração residual alvo de 4-6 ng/mL). Pacientes com contraindicações ao uso de imTOR ou, em casos especiais, a critério da equipe assistente - como em glomerulopatias - receberam micofenolato sódico na dose inicial de 1440mg/dia, com posterior ajuste a depender da ocorrência de eventos adversos. A azatioprina não é utilizada como parte do regime imunossupressor inicial do centro.

Corticoides por via endovenosa (metilprednisolona) ou oral (prednisona ou prednisolona) foram administrados apenas como pré-medicação antes das infusões de rATG, conforme descrito anteriormente, e nenhuma dose adicional foi feita após esse período com o objetivo de compor o regime de imunossupressão de manutenção.

Não houve uma rotina predeterminada de monitoramento dos pacientes, incluindo biópsias protocolares ou indicação rotineira de solicitação de pesquisa de DSA. Da mesma forma, não foi adotado um protocolo predefinido para introdução de corticoides após o TxR, sendo essa decisão atribuída ao médico assistente, com base em sua percepção e ponderação de risco e benefício. Também não houve conversões preemptivas da imunossupressão, sendo todas motivadas por preocupações relativas à falha de eficácia ou segurança do regime, em um cenário de vida real, com condutas fundamentadas na percepção da equipe clínica assistente.

### Definições

Função tardia do enxerto (DGF) foi definida como a necessidade de diálise na primeira semana pós-transplante, considerando e desconsiderando sessões isoladas realizadas dentro das primeiras 24h após o transplante, por hipercalemia e/ou hipervolemia^14^. A duração da DGF foi avaliada como o intervalo, em dias, entre o TxR e a última sessão de diálise.

Doador de critério expandido (DCE) foi definido como aquele com idade acima de 60 anos; ou com idade entre 50 e 59 anos e, no mínimo, dois critérios dentre os três a seguir: história de hipertensão arterial, acidente vascular cerebral como causa do óbito ou creatinina final superior a 1,5mg/dL^15^.

Diabetes mellitus pós-transplante (DMPT) foi definido como a necessidade de tratamento com antidiabéticos orais ou insulina após o TxR, em pacientes sem diagnóstico prévio de diabetes^16^.

O tratamento com estatinas foi iniciado de acordo com o julgamento médico, baseado nos valores de lípides e risco cardiovascular, sem que houvesse um protocolo pré-estabelecido para sua prescrição.

Rejeição aguda (RA) tratada foi definida como episódio de disfunção do enxerto que resultou em tratamento com pulsoterapia de metilprednisolona ou rATG, sessões de plasmaferese e/ou imunoglobulina, independentemente da realização ou do achado da biópsia. Rejeição aguda comprovada por biópsia (RACB) foi definida como episódio de RA cuja biópsia evidenciou rejeição mediada por anticorpos (AMR) ou rejeição aguda celular IA ou superior, de acordo com a classificação de Banff vigente no momento da biópsia^17^.

A função renal foi avaliada por meio da creatinina sérica e da taxa de filtração glomerular estimada (TFGe), calculada pela fórmula de CKD-EPI 2021^18^.

Agrupamos sob o termo “percepção de ineficácia terapêutica” - como motivo para introdução de corticoide - os episódios de RA tratada, o surgimento de DSA *de novo* ou o aumento do PRA durante o seguimento.

### Análise Estatística

As variáveis categóricas foram descritas por meio de frequências absolutas e porcentagens e comparadas utilizando-se o teste Qui-Quadrado ou o teste exato de Fisher, quando aplicável. O teste de Shapiro-Wilk foi utilizado para verificar a normalidade de distribuição das variáveis numéricas. As variáveis numéricas com distribuição normal foram expressas como média e desvio padrão; aquelas com distribuição não paramétrica foram expressas como mediana e intervalo interquartil (IIQ). As curvas de sobrevida foram construídas pelo método de Kaplan Meier. A sobrevida do enxerto teve o óbito censurado na análise.

Na análise da taxa de filtração glomerular estimada (TFGe), os dados faltosos foram imputados pelo método *Last Observation Carried Forward* (LOCF), atribuindo-se valor zero para pacientes que perderam o enxerto e o último valor disponível para aqueles que foram à óbito ou perderam o seguimento^19^. Para as demais variáveis numéricas e categóricas, nenhuma imputação de valores faltosos foi realizada.

A análise multivariada para identificar fatores de risco independentes para rejeição foi realizada utilizando a regressão de Cox. As variáveis com p-valor <0,1 na análise univariada foram incluídas no modelo multivariado. Tanto na análise multivariada quanto nas demais análises, foram considerados significativos os resultados com p-valor < 0,05.

### Aspectos Éticos

O estudo seguiu a Resolução 466/12 do Conselho Nacional de Saúde para pesquisas envolvendo seres humanos, tendo sido submetido à apreciação do Comitê de Ética em Pesquisa do Hospital Geral de Fortaleza e aprovado conforme parecer consubstanciado nº 5.486.193 e CAAE nº 59301622.0000.5040.

## Resultados

### Amostra e Características Clínico-Demográficas

Dos 1.192 transplantes renais realizados no período do estudo, 714 preenchiam inicialmente os critérios de inclusão. Após a aplicação dos critérios de exclusão, incluindo 4 óbitos ou perdas de enxerto por complicações cirúrgicas na primeira semana e 77 perdas precoces de seguimento, 562 pacientes foram incluídos na análise final (Figura 1).

**Figura 1:** Definição da amostra.

Abreviaturas – DSA: *anticorpo anti-HLA específico contra o doador*; HLA: Antígeno leucocitário humano; IS: imunossupressor; PRA: painel de reatividade de anticorpos; rATG: globulina antitimócito; TxR: transplante renal.

Os pacientes eram predominantemente do sexo masculino (71,4%) e pardos (74,6%), com mediana de idade de 48,1 anos (IIQ 35,5–58,6). A principal etiologia da doença renal foi indeterminada (38,1%), seguida por diabetes (19,6%) e glomerulopatias (14,1%). A quase totalidade dos TxR foi realizada com doador falecido (95,2%), sendo apenas 6,6% provenientes de DCE. A Tabela 1 detalha as características demográficas e clínicas dos pacientes no momento do TxR.

**Tabela 1:** Características demográficas e clínicas da amostra

Variável	N	Resultado
Idade do receptor (anos), mediana (IIQ)	562	48,1 (35,5–58,6)
Sexo masculino, n(%)	562	401 (71,4)
Raça, n(%) *Pardo* *Branco* *Preto* *Índio* *Amarelo*	562	419 (74,6) 84 (14,9) 57 (10,1) 1 (0,2) 1 (0,2)
IMC (Kg/m^2^), mediana (IIQ)	559	24,5 (22,0–27,7)
Etiologia da DRC, n(%) *Indeterminada* *DM* *GNC* *HAS* *DRPAD* *Urológica* *Outros*	562	214 (38,1) 110 (19,6) 79 (14,1) 62 (11,0) 48 (8,5) 26 (4,6) 23 (4,1)
Antecedente de DM, n(%)	562	118 (21,0)
Tempo em diálise (meses), mediana (IIQ)	561	32 (18–59)
Sorologia positiva, n(%) *Hepatite B* *Hepatite C* *HIV*	562	5 (0,9) 6 (1,1) 4 (0,7)
PRA I (%), mediana (IIQ) PRA I 1 – 50%, n(%)	562	0 (0–0) 89 (15,8)
PRA II (%), mediana (IIQ) PRA II 1 - 50%, n(%)	562	0 (0–0) 29 (5,2)
Tipo doador, n(%) *Falecido* *Vivo HLA haploidêntico* *Vivo HLA distinto*	562	535 (95,2) 14 (2,5) 13 (2,3)
Idade do doador, mediana (IIQ) DCE, n(%)*	535 535	32 (23–42) 37 (6,6)
Máquina perfusão, n(%)*	535	271 (48,2)
MM HLA, mediana (IIQ)*	535	3 (2–4)
MMDR, n(%) * *0* *1* *2*	535	197 (35,1) 261 (46,4) 77 (13,7)
TIF (h), mediana (IIQ)*	530	22,0 (18,7–26,0)

Abreviaturas – IIQ: Intervalo interquartílico; IMC: Índice de massa corporal; DRC: Doença renal crônica; DM: *Diabetes mellitus;* GNC: Glomerulonefrite crônica; HAS: Hipertensão arterial sistêmica; DRPAD: Doença renal policística autossômica dominante; DCE: Doador de critério expandido; MM: *Mismatch;* TIF: Tempo de isquemia fria.

### Imunossupressão

A dose mediana de timoglobulina utilizada foi de 5,5 mg/kg (IIQ 4,3–6,0). Ao longo do período do estudo, a dose total de rATG variou entre 3 mg/kg e 6 mg/kg, conforme alterações protocolares e participação em ensaios clínicos, sendo a dose de 4,5 mg/kg a mais recentemente padronizada para este grupo de risco.

Quanto aos regimes de manutenção, 44,5% dos pacientes receberam a combinação de tacrolimo e everolimo, 37,7% tacrolimo e sirolimo e 17,8% tacrolimo e micofenolato. As concentrações de tacrolimo e dos imTOR, bem como a dose mediana de micofenolato ao longo do seguimento, estão ilustradas na Figura 2.

**Figura 2:** Exposição aos imunossupressores no período de 3 anos.

Abreviaturas – C0: concentração sanguínea residual; EVR: everolimo; MPA: micofenolato; SRL: sirolimo; TAC: tacrolimo. Notas - (A) Concentração sanguínea residual do tacrolimo (ng/mL); Concentração sanguínea residual de sirolimo e everolimo (ng/mL) e dose média de micofenolato (mg/dia).

### Desfechos de Segurança, Efetividade, Função Renal e Sobrevidas

Dentre os desfechos de segurança investigados, a incidência de DMPT foi observada em 21,3% dos pacientes, e 4,7% necessitaram de tratamento com insulina. Mais da metade dos pacientes (54,9%) estava em uso de estatina ao final de 3 anos, e a mediana de ganho ponderal foi de 3,5 kg (IIQ -0,5 – +7,6).

Dentre os transplantes com doador falecido, 42,3% apresentaram DGF, com tempo mediano de duração de 8 dias (IIQ 3-17). Quando excluídas as sessões isoladas de diálise realizadas no período perioperatório, a incidência foi de 35,5%.

A incidência de rejeição aguda tratada foi de 10,2%, com a maioria dos episódios tratados não tendo sido submetida à biópsia ou apresentando, quando realizada, infiltrados *borderline* ou limítrofes. O tempo mediano para a ocorrência do primeiro episódio de RA tratada foi de 26 dias (IIQ 12–156,5 dias). Quarenta e três dos 57 pacientes (75,4%) receberam metilprednisolona para o tratamento dos episódios de RA, 9 (15,8%) foram tratados com rATG e 5 (8,8%) com plasmaferese e/ou imunoglobulina.

Considerando apenas as rejeições comprovadas por biópsia, a incidência foi de 3,2%, sendo 8 dos 18 episódios caracterizados como rejeições celulares leves, com tempo mediano de ocorrência de 39 dias (IIQ 13,75–170,5 dias).

As sobrevidas do paciente e do enxerto, ao final de 3 anos de seguimento, foram de 94,1% e 94,9%, respectivamente. A Tabela 2 detalha os desfechos em 3 anos.

**Tabela 2:** Desfechos de segurança e efetividade em 3 anos

Variável	N	Resultado
DMPT, n(%)	423	90 (21,3)
DMPT com necessidade de insulina, n(%)	423	20 (4,7)
Necessidade de estatinas, n(%)	532	292 (54,9)
Ganho de peso (Kg), mediana (IIQ)	478	+3,5 (–0,5 – +7,6)
DGF, n(%)*	532	225 (42,3)
DGF sem considerar diálise isolada POI/1o. PO, n(%)*	532	189 (35,5)
Tempo em DGF (dias), mediana (IIQ)*	224	8 (3–17)
RA tratada, n(%)	559	57 (10,2)
*BxR não realizada* *Sem RA* *Infiltrado borderline* *Banff IA* *Banff IB* *Banff IIA* *Banff AMR*		18 1 20 8 2 5 3
RACB, n(%) RACB + infiltrados *borderline*, n(%)	559 559	18 (3,2) 38 (6,8)
Sobrevida livre de RA tratada em 3 anos, n(%)	560	454 (89,6)
Sobrevida livre de RACB em 3 anos, n(%)	560	488 (96,7)
Perda do enxerto, n(%) *IFTA não imune* *Rejeição aguda* *Não funcionamento primário de enxerto* *Trombose vascular* *Complicação pós biópsia* *Fístula urinária* *GN recorrente ou de novo* *Pseudoanerisma de artéria renal* *Necrose cortical* *Nefropatia por BKV* *Pielonefrite aguda* *Infecção da loja renal*	562	28 (5,0) 5 3 3 3 3 3 2 2 1 1 1 1
Sobrevida do enxerto em 3 anos, n(%)	562	502 (94,9)
Óbito, n(%) *Infecção* *Neoplasia* *Evento cardiovascular* *Pancreatite* *Choque hemorrágico* *Causa desconhecida*	562	32 (5,7) 20 4 4 2 1 1
Sobrevida do paciente em 3 anos, n(%)	562	501 (94,1)
TFGe em 3 anos (mL/min/1,73m^2^), mediana (IIQ)	500	65,0 (49,5–82,7)
TFGe em 3 anos com ajuste LOCF (mL/min/1,73m^2^), mediana (IIQ)	561	62,0 (46,4–81,1)

Abreviaturas – DMPT: *Diabetes mellitus* pós-transplante; IIQ: Intervalo interquartílico; DGF: *Delayed graft function;* RA: Rejeição aguda; BxR: Biópsia renal; RACB: Rejeição aguda comprovada por biópsia; IFTA: Fibrose intersticial e atrofia tubular; GN: Glomerulonefrite; TFG: Taxa de filtração glomerular; LOCF: *Last observation carried foward.*

Em análise multivariada, a idade do receptor (HR 0,946; IC95% 0,923–0,969; p < 0,001) e as incompatibilidades HLA (HR 1,312; IC95% 1,021–1,687; p = 0,034) foram as variáveis associadas, de forma independente, à ocorrência de RACB (aqui incluídos os infiltrados *borderline*) (Tabela 3).

**Tabela 3:** Fatores de risco para rejeição aguda comprovada por biópsia em 3 anos

	Univariada			Multivariada N = 35*		
	HR	IC 95%	P valor	HR	IC 95%	P valor
Idade do receptor (anos)	0,953	0,931–0,976	<0,001	**0,946**	**0,923–0,969**	**<0,001**
Sexo masculino	0,771	0,394–1,507	0,447	NA		
Raça negra	1,366	0,533–3,499	0,516	NA		
IMC (Kg/m2)	0,928	0,858–1,005	0,065	0,984	0,960-1,069	0,709
Antecedente de DM	0,205	0,049–0,853	0,029	0,351	0,081-1,522	0,162
Tempo em diálise (meses)	1,003	0,996–1,010	0,388	NA		
Doador falecido	0,562	0,173–1,828	0,338	NA		
Idade do doador (anos)	1,528	0,763–3,060	0,232	NA		
MM HLA	1,368	1,045–1,790	0,022	**1,312**	**1,021–1,687**	**0,034**
PRA I (%)	1,021	0,993–1,050	0,145	NA		
PRA II (%)	0,993	0,932–1,059	0,839	NA		
Dose rATG (mg/Kg)	0,769	0,597–0,992	0,044	0,876	0,673-1,142	0,328
IS manutenção inicial com imTOR (vs. MPA)	0,963	0,424–2,186	0,963	NA		
DGF desconsiderando diálise isolada no POI/1º. PO	1,458	0,746–2,848	0,270	NA		

Abreviaturas – IMC: Índice de massa corporal; DM: *Diabetes mellitus*; MM: *Mismatch*; ATG: Globulina antitimócito; IS: Imunossupressão; MPA: Micofenolato; DGF: *Delayed graft function;* PO: Pós-operatório.

### Introdução do Corticoide

Ao longo do seguimento, 71 pacientes (12,6%) demandaram a introdução do corticoide ao regime de manutenção, sendo que a maioria das introduções ocorreu nos primeiros meses após o TxR, com mediana de 125,5 dias (IIQ 31,7–409). Os principais motivos foram a percepção de ineficácia terapêutica do regime e a composição em regime imunossupressor considerado de menor eficácia (Figura 3). Entre os 40 casos considerados como percepção de falha de eficácia do regime, 34 corresponderam a episódios de rejeição aguda tratada, 5 ao surgimento de DSA e 1 caso ao aumento dos valores de PRA, sem DSA detectável.

**Figura 3:** Necessidade de introdução de corticoide ao longo dos 3 anos de acompanhamento.

Dos 57 pacientes com RA tratada, 22 não iniciaram corticoide após o episódio. Em 18 desses casos, não houve comprovação da rejeição na biópsia (8 infiltrados *borderline* e 10 não biopsiados). Dentre os 4 pacientes com RACB, 1 perdeu o enxerto em decorrência do episódio de rejeição e 1 por complicação cirúrgica precoce, impossibilitando a introdução posterior do corticoide. Nos outros 2 pacientes com RACB, o esteroide não foi iniciado e não foi possível, a partir dos registros, identificar o motivo.

Dos 13 casos em que o corticoide foi introduzido para compor um regime de menor eficácia, em 5 a combinação foi tacrolimo associado à azatioprina, 3 correspondiam a regimes com tacrolimo isolado, 1 a regime com micofenolato isolado e, em 4 deles, o corticoide foi mantido como o único imunossupressor.

## Discussão

Este estudo demonstrou que o uso de regimes imunossupressores livres de esteroides, baseados na indução com rATG e na terapia de manutenção inicial com tacrolimo e micofenolato ou imTOR, foi eficaz na prevenção de episódios de rejeição aguda e apresentou bom perfil de segurança no acompanhamento de 3 anos.

Esses achados estão em consonância com estudos uni e multicêntricos previamente publicados, que demonstram que regimes livres de esteroides são eficazes em pacientes de baixo a moderado risco imunológico quando se utilizam terapias de manutenção de alta eficácia, ou seja, a combinação de tacrolimo e micofenolato ou imTOR e indução com rATG^20-23^. Nossos dados acrescentam informações sobre um subgrupo pouco explorado na literatura, que é o de pacientes em uso de tacrolimo associado a um imTOR.

Adicionalmente, embora não tenhamos realizado uma comparação direta dos desfechos entre os subgrupos que utilizaram imTOR versus micofenolato, nas análises multivariadas, o tipo de antiproliferativo inicial não emergiu como um preditor independente para a ocorrência de RACB.

Digno de nota, um dos desafios para a implementação rotineira da estratégia livre de esteroides é estabelecer quais são as doses e concentrações-alvo dos fármacos capazes de garantir eficácia, minimizando os eventos adversos, notadamente a nefrotoxicidade. Nesse sentido, a demografia de nossa amostra provavelmente constitui um fator crucial para garantir os bons resultados observados, uma vez que receptores de transplantes de doadores jovens são menos suscetíveis aos eventos adversos relacionados à super ou subexposição aos fármacos imunossupressores^24^.

Outro desafio é identificar quais pacientes claramente se beneficiam dessa estratégia e em quais ela deve ser evitada. A incidência de DMPT, o uso de estatinas e o ganho ponderal foram semelhantes aos observados em coortes que utilizam esteroides^25-28^. A ausência de um grupo controle submetido à imunossupressão com esteroides não nos permitiu identificar se a estratégia trouxe benefícios reais à amostra. Outros eventos adversos associados ao uso crônico de esteroides, como catarata, osteoporose, infecções e eventos cardiovasculares não fatais, não foram avaliados.

Chama atenção a baixa taxa de rejeição, ainda que considerando todos os episódios tratados, com ou sem confirmação por biópsia, bem como a boa função renal e as baixas taxas de perda do enxerto e de óbito em 3 anos. Ressalta-se que se trata de uma coorte de pacientes de baixo risco imunológico, jovens e que receberam predominantemente rins de doadores de critério padrão, quase metade deles perfundidos em máquina de perfusão – fator evidenciado pela incidência de DGF significativamente inferior à média nacional^29^. Dentro desse subgrupo de baixo risco imunológico, a análise multivariada indica que pacientes mais jovens e com maior número de incompatibilidades HLA apresentam risco mais elevado de rejeição. Igualmente, a ausência de um grupo controle em uso de corticoide nos impede de avançar em conclusões mais sólidas sobre a eficácia do regime. Também não está claro até que ponto é possível avançar dentro do grupo de pacientes de maior risco imunológico.

Destaca-se, ainda, o fato de 71% dos pacientes serem do sexo masculino. Evidências nacionais e locais apontam para a inequidade de acesso ao transplante entre mulheres^30^. Entretanto, esse percentual de homens excede o descrito em coortes prévias de nossa região (cerca de 60%)^31,32^, o que pode estar relacionado ao maior percentual de mulheres sensibilizadas ou com doenças autoimunes que requerem uso basal de esteroides^33^.

Um baixo percentual de pacientes (12,6%) teve o corticoide introduzido ao regime ao longo dos anos, o que ocorreu dentro dos primeiros meses, sendo a maioria dos casos motivada por preocupações com a eficácia - fator que reflete o principal motivo da hesitação dos serviços em implantar a estratégia de forma rotineira.

Este estudo apresenta algumas limitações que devem ser apontadas. Primordialmente, a ausência de um grupo controle impede conclusões definitivas sobre a eficácia e a segurança da estratégia. Uma vez que o regime livre de esteroides é a prática adotada no centro para pacientes de baixo a moderado risco imunológico, não dispomos de um grupo controle comparável do ponto de vista demográfico e clínico. A utilização de um grupo controle proveniente de outro centro poderia introduzir vieses significativos, em virtude das diferenças de demografia e de práticas clínicas. Além disso, trata-se de um estudo realizado em um centro único, localizado em uma região cuja demografia dos transplantes é peculiar, com baixa taxa de utilização de doadores de critérios expandidos e incidência de DGF abaixo da média nacional. A exclusão de pacientes que perderam o enxerto ou morreram na primeira semana do transplante pode, eventualmente, ter adicionado algum viés de seleção. Não dispomos de informações sobre o acompanhamento regular do PRA, e não foram realizadas biópsias protocolares. Também não exploramos se essa simplificação do regime poderia aumentar a aderência aos imunossupressores. Apesar dessas limitações, este estudo apresenta dados de vida real, com uma amostra robusta, e descreve os resultados de uma experiência pioneira no país e na América Latina, além de agregar informações sobre a estratégia livre de corticoides em pacientes em uso de tacrolimo e imTOR, para a qual as evidências ainda são escassas.

Em conclusão, nessa coorte de pacientes de baixo a moderado risco imunológico, o regime de manutenção livre de corticoides demonstrou bom perfil de efetividade e segurança ao final de 3 anos após o TxR.

## Data Availability

O banco de dados que dá suporte aos resultados deste estudo está disponível mediante solicitação fundamentada ao autor correspondente, Taina Veras de Sandes-Freitas.
